# Short- and Long-term Effects of Bacterial Gastrointestinal Infections

**DOI:** 10.3201/eid1401.070524

**Published:** 2008-01

**Authors:** Anders Ternhag, Anna Törner, Åke Svensson, Karl Ekdahl, Johan Giesecke

**Affiliations:** * Karolinska Institute, Stockholm, Sweden; † Swedish Institute for Infectious Disease Control, Solna, Sweden; ‡ Stockholm University, Stockholm, Sweden; § European Centre for Disease Prevention and Control, Stockholm, Sweden

**Keywords:** Gastroenteritis, campylobacter infections, enterobacteriaceae infections, epidemiology, registries, risk, hospitalization, statistics, numerical data, research

## Abstract

During 1997–2004, microbiologically confirmed gastrointestinal infections were reported for 101,855 patients in Sweden. Among patients who had *Salmonella* infection (n = 34,664), we found an increased risk for aortic aneurysm (standardized incidence ratio [SIR] 6.4, 95% confidence interval [CI] 3.1–11.8) within 3 months after infection and an elevated risk for ulcerative colitis (SIR 3.2, 95% CI 2.2–4.6) within 1 year after infection. We also found this elevated risk for ulcerative colitis among *Campylobacter* infections (n = 57,425; SIR 2.8, 95% CI 2.0–3.8). Within 1 year, we found an increased risk for reactive arthritis among patients with *Yersinia* enteritis (n = 5,133; SIR 47.0, 95% CI 21.5–89.2), *Salmonella* infection (SIR 18.2, 95% CI 12.0–26.5), and *Campylobacter* infection (SIR 6.3, 95% CI 3.5–10.4). Acute gastroenteritis is sometimes associated with disease manifestations from several organ systems that may require hospitalization of patients.

## Abstract 2

Bacterial gastrointestinal infections are associated with short- and long-term complications from several organ systems.

Bacterial gastrointestinal infections continue to cause illness and death and contribute to economic loss in most parts of the world, including high-income countries that have developed surveillance and control programs. The symptoms of acute bacterial intestinal infection are usually mild to moderate, and spontaneous remission occurs (*1**)*, but in some cases, the disease can cause rapid deterioration of a patient’s condition.

An episode of acute enteric infection involving extraintestinal organs can also lead to complications and trigger chronic disease. Complications include irritable bowel syndrome (*2**)*, reactive arthritis (*3**)*, hemolytic uremic syndrome (HUS) (*4**)*, and Guillain-Barré syndrome (GBS) (*5*). There may be other, perhaps unusual and less documented, late effects of acute enteric infections, such as inflammatory bowel disease (*6**)*.

In Sweden, there is no active follow-up on reported cases of bacterial enteric infection in terms of disease outcome or long-term complications. During the 8-year period 1997–2004, >100,000 persons with acute gastrointestinal infection were reported within the national surveillance program for communicable diseases. We present a retrospective cohort study of these patients to investigate the association between exposure to a bacterial pathogen and the risk for autoimmune illness, gastrointestinal complications, and extraintestinal infectious disease.

## Materials and Methods

Participants comprised persons with intestinal infection (nontyphoidal *Salmonella* spp., *Campylobacter* spp., *Yersinia enterocolitica*, *Shigella* spp., or enterohemorrhagic *Escherichia coli* [EHEC]) reported to the Swedish Institute for Infectious Disease Control during 1997–2004. We collected data on age, sex, date reported, and country of infection and used social security numbers for identification. This identification number was used to link our cohort of cases (those with short-term complications occurring within 3 months or long-term effects within 1 year after infection) to the Swedish Hospital Discharge (covers all hospital in Sweden) and Causes of Death registers. Ethics permission was obtained from the Ethical Committee, Karolinska Institute. Discharge diagnoses must be reported to the register; therefore, any study using this register is, in practice, population based. The Hospital Discharge Register was validated by using a diagnosis of acute myocardial infarction; underreporting was <1%, main diagnosis was missing for <1% of cases, and correct diagnosis was made for 86% (*7**)*.

We calculated the follow-up time for each case as person-time from reported date of infection to an event, death, or study termination. Person-years were then compared with a Swedish standard population of 5-year age groups to calculate the expected number of cases for each disease. Standardized incidence ratios (SIRs) were constructed by dividing the observed number of cases with the expected number of cases. Ninety-five percent exact confidence intervals (CIs) were calculated under the assumption that the number of observed cases were Poisson distributed. CIs that do not overlap 1 indicate that the number of observed cases is significantly different from the number of cases expected in a population cohort of similar age and sex distribution. The described method is called indirect standardization, and interpretation of results is similar to relative risk interpretation, i.e., comparing the risk for disease in an exposed cohort to the risk for disease in an unexposed cohort.

We previously estimated standardized mortality ratios (SMRs) for *Salmonella* (*8**)* and *Campylobacter* infections (*9**)* and showed that country of infection (domestic or abroad) was an effect modifier; i.e., the SMR differed substantially between these 2 strata and no pooled SMR could be calculated. The underlying factor for this interaction was probably that the term *abroad* served as a proxy for healthiness or a healthy traveler effect. For our present analysis, we divided the cohort into 2 strata on the basis of country of infection (Sweden or abroad), but no statistical significant interaction was evident. We concluded that crude SIRs irrespective of country of infection could be estimated. All analyses were conducted by using SAS statistical software, version 8.2 (SAS Institute, Inc., Cary, NC, USA).

## Results

Demographic data on the 101,855 study participants and frequency counts for infectious agents are summarized in Table 1. *Campylobacter* spp. caused the most cases, 57,425 (56%). The second most frequent pathogen was *Salmonella* spp., the causative agent in 34,664 cases (34%); distribution of serovars is shown in Table 2. Of all cases of gastroenteritis, *Yersinia* spp. accounted for 5,133 (5%) cases; *Shigella* spp. 3,813 (4%); and EHEC 820 (<1%).

**Table 1:** Distribution of infectious agents, age and sex for 101,855 study participants, Sweden, 1997−2004

Characteristic	Nontyphoid *Salmonella* spp.	*Campylobacter* spp.	*Shigella* spp.	EHEC*	*Yersinia* spp.
No. participants					
Female	17,524	27,067	2,145	451	2,390
Male	17,140	30,358	1,668	369	2,743
Mean age, y (range)					
Female	37 (0−100)	37 (0−99)	33 (1−89)	25 (0−98)	28 (0−95)
Male	36 (0−97)	37 (0−98)	33 (0−83)	19 (0−85)	27 (0−94)

*EHEC, enterohemorrhagic *Escherichia coli*.

**Table 2:** Most frequent serotypes isolated among study participants with nontyphoid *Salmonella* infection, Sweden, 1997−2004

*Salmonella* serotype	Frequency	Relative frequency, %
*S*. species, not subtyped	14,643	42
*S.* Enteritidis	10,580	31
*S*. Typhimurium	2,607	8
*S.* Virchow	741	2
*S.* Hadar	734	2
Other specified serotypes	5,359	15
Total	34,664	100

Table 3 shows the number of reported case-patients with specific diseases within 3 months of an episode of bacterial gastrointestinal infection, along with expected number of cases and SIRs. Not surprisingly, the highest risks were found for HUS after EHEC infection and GBS following campylobacter infection. Although SIRs were quite elevated, absolute risks were more moderate; among 820 cases of EHEC infection, we found 13 episodes of HUS (1.6%), 57,425 cases of campylobacteriosis, 13 cases of GBS (0.02%), 5,133 cases of *Yersinia* infection, and 9 cases of reactive arthritis (0.2%). The risk for aortic aneurysm among patients with salmonellosis was significantly higher than expected (SIR 6.4, 95% CI 3.1–11.8). The absolute risk for bacteremia/sepsis was 0.02% for case-patients with *Campylobacter* infection and 0.03% for those with salmonellosis. For many complications, we did not find any statistically significant elevated risks. Other complications that we had hypothesized to be associated with gastrointestinal infections could not be shown. Only a few cases were found within 3 months, contributing to imprecise estimates of SIRs.

**Table 3:** Complications associated with gastroenteritis, 3 months postinfection, among 101,855 patients with bacterial gastrointestinal infection, Sweden, 1997−2004*

Disease	Infecting organism	Obs	Exp	SIR	95% CI
Respiratory system					
Bacterial pneumonia, pneumonitis due to food and vomit	Nontyphoid *Salmonella* spp.	24	13.5	1.8	1.1–2.6
	*Campylobacter* spp.	17	21.4	0.8	0.5–1.3
	EHEC	1	0.3	3.1	0.1–17.2
	*Shigella* spp.	1	1.1	0.9	0.02–5.2
	*Yersinia* spp.	4	2.3	1.8	0.5–4.5
Blood					
Hemolytic-uremic syndrome	Nontyphoid *Salmonella* spp.	1	<0.05	55.5	1.4–309.1
	*Campylobacter* spp.	2	<0.05	81.0	9.8–292.7
	EHEC	13	<0.05	18,333.4	9,761.8–31,350.6
Circulatory system					
Aortic aneurysm	Nontyphoid *Salmonella* spp.	10	1.6	6.4	3.1–11.8
	*Campylobacter* spp.	5	2.4	2.06	0.7–4.8
	*Yersinia* spp.	1	0.2	5.2	0.1–28.9
Endocarditis	Nontyphoid *Salmonella* spp.	2	0.4	5.7	0.7–20.5
Digestive system					
Peritonitis	Nontyphoid *Salmonella* spp.	1	0.6	1.9	0.05–10.1
	*Campylobacter* spp.	2	0.9	2.3	0.4–8.4
Perforation of intestine (nontraumatic)	Nontyphoid *Salmonella* spp.	1	0.1	9.7	0.3–54.0
	*Campylobacter* spp.	2	0.2	12.39	1.5–44.7
	EHEC	1	<0.05	655.3	16.6–3,651.0
Idiopathic acute pancreatitis	Nontyphoid *Salmonella* spp.	6	2.6	2.3	0.9–5.1
	*Campylobacter* spp.	7	4.1	1.7	0.68–3.5
Hepatic failure	Nontyphoid *Salmonella* spp.	1	0.3	4.0	0.1–22.2
Infectious diseases					
Septicemia	Nontyphoid *Salmonella* spp.	10	2.6	3.9	1.8–7.1
	*Campylobacter* spp.	14	4.1	3.4	1.9–5.7
	*Shigella* spp.	1	0.2	5.1	0.1–28.2
Nervous system					
Guillain-Barré syndrome	*Campylobacter* spp.	13	0.2	66.6	35.5–114.0
Musculoskeletal system					
Pyogenic arthritis	Nontyphoid *Salmonella* spp.	4	0.8	5.2	1.4–13.4
	*Yersinia* spp.	1	0.1	10.1	0.3–56.2
Osteomyelitis	Nontyphoid *Salmonella* spp.	3	0.6	5.4	1.1–15.7

*Obs, observed number of cases; Exp, expected number of cases; SIR, standardized incidence ratio; CI, confidence interval; EHEC, enterohemorrhagic *Escherichia coli*.

Within 1 year of acute bacterial gastrointestinal infection, case-patients with *Yersinia* enteritis were at increased risk for reactive arthritis (SIR 47.0, 95% CI 21.5–89.2), *Salmonella* infection (SIR 18.2, 95% CI 12.0–26.5), and *Campylobacter* infection (SIR 6.3, 95% CI 3.5–10.4) (Table 4). The risk for ulcerative colitis was elevated among patients with salmonellosis (SIR 3.2, 95% CI 2.2–4.6) and, to a lesser extent, among patients with campylobacteriosis (SIR 2.8, 95% CI 2.0–3.8). Of the 29 patients in our salmonellosis cohort who had ulcerative colitis, 13 (44%) had first experienced ulcerative colitis during the 10-year period before the acute infection. Among patients with campylobacteriosis, we found 42 with ulcerative colitis, of whom 18 (43%) had received a diagnosis of ulcerative colitis in the 10-year period before the infection. We did not find any increased risk for Crohn’s disease in the same group of patients. We did not find any elevated risk for many of the rheumatologic diseases included in the present study in any of the participants. The distribution of *Salmonella* serotypes among patients with aortic aneurysm, reactive arthritis, and ulcerative colitis in our cohort did not differ in any substantial way from the whole salmonellosis cohort (Table 5), although the number of patients was rather small.

**Table 4:** Complications associated with gastroenteritis, 1 year postinfection, among 101,855 patients with bacterial gastrointestinal infection, Sweden, 1997−2004*

Disease	Infecting organism	Obs	Exp	SIR	95% CI
Digestive system					
Crohn’s disease	*Campylobacter* spp.	27	17.1	1.6	1.0–2.3
	*Salmonella* spp.	14	10.3	1.4	0.8–2.3
	*Shigella* spp.	1	1.1	0.9	0.02–5.2
	*Yersinia* spp.	2	1.1	1.8	0.2–6.4
Ulcerative colitis	*Campylobacter* spp.	42	14.8	2.8	2.0–3.8
	EHEC	1	0.1	6.8	0.2–37.7
	*Salmonella* spp.	29	9	3.2	2.2–4.6
	*Yersinia* spp.	3	1	2.9	0.6–8.5
Other specified/unspecified noninfective gastroenteritis and colitis	*Campylobacter* spp.	37	14.9	2.5	1.8–3.4
	*Salmonella* spp.	30	9.2	3.3	2.2–4.6
	*Yersinia* spp.	10	1.3	7.6	3.7–14.0
Irritable bowel syndrome	*Campylobacter* spp.	15	5	3.0	1.7–5.0
	*Salmonella* spp.	5	3	1.7	0.5–3.9
	*Yersinia* spp.	3	0.4	7.8	1.6–22.9
Intestinal malabsorption	*Salmonella* spp.	1	0.6	1.7	0.04–9.3
	*Yersinia* spp.	1	0.1	7.9	0.2–43.7
Musculoskeletal system					
Postdysenteric arthropathy, Reiter disease, other reactive arthropathies	*Campylobacter* spp.	15	2.4	6.3	3.5–10.4
	*Salmonella* spp.	27	1.5	18.2	12.0–26.5
	*Shigella* spp.	2	0.1	13.4	1.6–48.4
	*Yersinia* spp.	9	0.2	47.0	21.5–89.2
Rheumatoid arthritis	*Campylobacter* spp.	22	22.5	1.0	0.6–1.5
	EHEC	1	0.2	5.8	0.2–32.1
	*Salmonella* spp.	9	14.7	0.6	0.3–1.2
	*Shigella* spp.	1	1.2	0.8	0.02–4.7
	*Yersinia* spp.	3	1.5	2.0	0.4–5.7
Other arthritis	*Campylobacter* spp.	8	3.8	2.1	0.9–4.2
	*Salmonella* spp.	4	2.5	1.6	0.4–4.1
	*Shigella* spp.	1	0.2	4.3	0.1–24.1
	*Yersinia* spp.	1	0.4	2.4	0.06–13.4
Other necrotizing vasculopathies (Goodpasture syndrome, TTP, Wegener granulomatosis, giant cell arteritis)	*Campylobacter* spp.	10	3.3	3.1	1.5–5.6
	EHEC	0	<0.05	32.8	0.8–183.0
	*Salmonella* spp.	1	2.1	0.5	0.01–2.7
Systemic lupus erythematosus	*Campylobacter* spp.	5	3.4	1.5	0.5–3.4
	*Salmonella* spp.	2	2.1	1.0	0.1–3.5
Systemic sclerosis	*Campylobacter* spp.	2	1.7	1.2	0.2–4.4
	*Salmonella* spp.	3	1.1	2.8	0.6–8.1
Other systemic involvement of connective tissue (Sjögren syndrome, mixed connective tissue disease, polymyalgia rheumatica)	*Campylobacter* spp.	12	5	2.4	1.2–4.2
	*Salmonella* spp.	4	3.1	1.3	0.4–3.3
	*Shigella* spp.	1	0.2	4.2	0.1–23.3
Ankylosing spondylitis	*Campylobacter* spp.	2	1.1	1.8	0.2–6.4
	*Salmonella* spp.	1	0.7	1.5	0.04–8.1

*Obs, observed number of cases; Exp, expected number of cases; SIR, standardized incidence ratio; CI, confidence interval; EHEC, enterohemorrhagic *Escherichia coli*.; TTP, thrombotic thrombocytopenic purpura.

**Table 5:** *Salmonella* serotypes among patients with aortic aneurysm, reactive arthropathies, and ulcerative colitis, Sweden, 1997−2004

Disease or condition	*Salmonella* serotype	Frequency	Relative frequency* (%)
Aortic aneurysm (n = 10)	*S.* Enteritidis	3	30 (31)
	*S.* Dublin	2	20 (<1)
	*S.* Virchow	1	10 (2)
	Other *S.* spp.	4	40 (42)
Postdysenteric arthropathy, Reiter disease, other reactive arthropathies (n = 27)	*S.* Enteritidis	10	37 (31)
	*S*. Typhimurium	3	11 (8)
	*S.* London	1	4 (<1)
	Other *S.* spp.	13	48 (42)
Ulcerative colitis (n = 29)	*S.* Enteritidis	7	24 (31)
	*S.* Typhimurium	4	14 (8)
	*S.* Kottbus	1	3 (<1)
	*S.* Agona	1	3 (1)
	*S.* Ituri	1	3 (<1)
	Other *S.* spp.	15	52 (42)

*Relative frequency in total cohort, n = 34,664.

## Discussion

Our data confirm the elevated risk for complications and long-term sequelae after an episode of acute bacterial gastroenteritis. We have presented new estimates of the absolute and relative risk for well-described complications such as HUS after EHEC infection, GBS after an episode of *Campylobacter* enteritis, and reactive arthritis after *Yersinia* enteritis. Another complication that we have been able to verify is aortic aneurysm after an episode of salmonellosis. Perhaps more unexpected, the risk for ulcerative colitis was elevated in the cohort of patients with salmonellosis and campylobacteriosis. The distribution of *Salmonella* serovars was the same among patients with and without complications. The finding of no major difference in the distribution of *Salmonella* serovars between the group of patients with and without complications indicates that factors other than *Salmonella* serovar alone determine the risk for complications.

Compared with other studies, our new estimate of the risk for HUS after EHEC infection is lower than previously reported (*10*,*11*). An explanation of our lower estimates could be that we used only International Classification of Diseases (ICD) codes specific for HUS. Several of these cases may in fact be classified under nonspecific ICD codes that also include a large proportion of cases unrelated to HUS. However, had we included them in the analysis, any association with the infections would have been diluted. Our estimate of risk for GBS and campylobacter are in line with a study in England that showed a risk of <2/10,000 that GBS will develop in a patient with campylobacteriosis (*12**)*. These results are also in line with a previous study in Sweden (*13**)*. All estimates of complications in this study are based on discharge data from the Hospital Discharge Register; this means that minor complications that either were not presented to any doctor or were handled only by general practitioners were not available for this analysis. At the population level, reactive rheumatologic symptoms associated with infection are typically mild and transient (*14**)*. This is probably the reason why our estimate of reactive arthritis after *Yersinia* infection is quite low, although similar low risks have been reported elsewhere (*15**)*.

In patients with atherosclerotic disease, or in those with preexisting aneurysms, transient bacteremia with nontyphoidal *Salmonella* infection can result in vascular infections (*16**–**18**)*. Most of these aneurysms described previously have been localized in the subrenal segment of the abdominal aorta (*17**)*. *Salmonella* spp. in these patients can invade the arterial intima and cause a localized endothelial infection that results in an aneurysm or the enlargement of a previously existing aneurysm. This may explain the association between *Salmonella* infection and aortic aneurysm in this study.

Our findings of an elevated risk for ulcerative colitis in the cohort of patients with salmonellosis and campylobacteriosis need further study. In another large cohort study, an association between acute gastroenteritis and inflammatory bowel disease was identified (n = 43,013), where the incidence rate for ulcerative colitis was 40 per 100,000 person-years, a doubling of the risk for those unexposed to infection (*19**)*. We do not know why an episode of infectious gastroenteritis could contribute to the initiation or exacerbation of ulcerative colitis. Seasonal variation in the onset of ulcerative colitis, and reports that excessive childhood infections are associated with higher risk for ulcerative colitis, may support the hypothesis that infections could be triggers of disease (*20**)*. From this study, we cannot say whether there is a causal relationship between *Salmonella* and *Campylobacter* infections and relapse of disease in patients with known ulcerative colitis, or whether the infection could trigger ulcerative colitis in susceptible persons. We cannot entirely rule out that the findings are an artifact, resulting from an increased number of medical examinations and stool cultures in a group of patients with diarrhea because of a known or unknown inflammatory bowel disease. More study is needed to confirm or refute our findings.

Because irritable bowel syndrome is diagnosed and treated at hospital in only a minority of patients, our estimates are probably too low. Many studies have not used a control group but reported only the numbers and percentages of patients who had irritable bowel syndrome after gastroenteritis (*21**)*; 1 study with controls estimated a relative risk of 11.9 (CI 6.7–21) after 1 year of follow-up (*22*).

Our study has some limitations. Perhaps the most serious one is the selection bias of patients entering the gastroenteritis cohort. Only a small fraction of all patients with *Salmonella* infection, for example, seek medical care, have a stool sample taken, and are eventually reported to national surveillance (*23*). This could have an effect on the results, especially if we are collecting data on those with the most severe disease; disease severity itself affects complications and sequelae. Another limitation is the lack of information on confounding factors among study participants, especially coexisting illnesses such as malignant disease or immunodeficiencies of any cause. Such coexisting illnesses could perhaps increase to some extent the risk for complications (*6**)*, but our results on the effect of disease from gastrointestinal infections would not have changed. Although the quality of the Swedish Hospital Discharge Register is quite good, there is always a general problem of reliability in registry-based epidemiologic research.

In conclusion, we studied the risk for complications 3 months and 1 year after acute bacterial gastroenteritis and found disease manifestations from several organ systems that required hospitalization of patients. These findings are a reminder of, and could be an argument for, the usefulness of existing control programs targeted to control bacterial enteric disease.
